# Proteins interacting with cloning scars: a source of false positive protein-protein interactions

**DOI:** 10.1038/srep08530

**Published:** 2015-02-23

**Authors:** Charles A. S. Banks, Gina Boanca, Zachary T. Lee, Laurence Florens, Michael P. Washburn

**Affiliations:** 1 Stowers Institute for Medical Research, Kansas City, MO 64110; 2 Departments of Pathology & Laboratory Medicine, University of Kansas Medical Center, Kansas City, KS 66160

## Abstract

A common approach for exploring the interactome, the network of protein-protein interactions in cells, uses a commercially available ORF library to express affinity tagged bait proteins; these can be expressed in cells and endogenous cellular proteins that copurify with the bait can be identified as putative interacting proteins using mass spectrometry. Control experiments can be used to limit false-positive results, but in many cases, there are still a surprising number of prey proteins that appear to copurify specifically with the bait. Here, we have identified one source of false-positive interactions in such studies. We have found that a combination of: 1) the variable sequence of the C-terminus of the bait with 2) a C-terminal valine “cloning scar” present in a commercially available ORF library, can in some cases create a peptide motif that results in the aberrant co-purification of endogenous cellular proteins. Control experiments may not identify false positives resulting from such artificial motifs, as aberrant binding depends on sequences that vary from one bait to another. It is possible that such cryptic protein binding might occur in other systems using affinity tagged proteins; this study highlights the importance of conducting careful follow-up studies where novel protein-protein interactions are suspected.

Recently, there has been a drive both to systematically define the protein content of cells (the proteome)^1^, and to map the interactions between these proteins (the interactome)^2^. Affinity purification coupled with mass spectrometry (AP-MS) is a common approach used to explore protein-protein interactions^3^. Many hundreds of endogenous cellular proteins may copurify with an affinity tagged bait. These might be present because of bona fide direct or indirect physical interactions that reflect genuine protein-protein interactions that occur in intact cells. Alternatively, proteins that do not interact with the endogenous counterpart of the bait in living cells might copurify with the tagged bait for a variety of other reasons^4^,^5^. Affinity tagged baits derived from commercially available ORFeome collections have been used in a number of studies aimed at mapping the network of protein-protein interactions in cells^6^,^7^,^8^; the recombinant proteins expressed using such systems are modified versions of the native protein with additional amino acid sequences for affinity tags, protease cleavage sites for tag removal, and in some cases additional amino acids resulting from cloning “scars”. Here we report a case in which a single valine, appended to the C terminus of bait proteins (a cloning scar), resulted in spurious interactions between some tagged bait proteins and endogenous prey proteins containing PDZ domains. Such false positive interactions were not apparent from control purifications expressing the tag alone; the interactions depend both on the sequence of the C terminal amino acids of the bait protein and the presence of the additional valine. This highlights one possible source of false positive protein-protein interactions from AP-MS data commonly used to develop protein-protein interaction networks.

## Results

### Using the Flexi®-format human ORFeome collection to express Halo-tagged bait proteins for AP-MS studies

**Figure 1:**
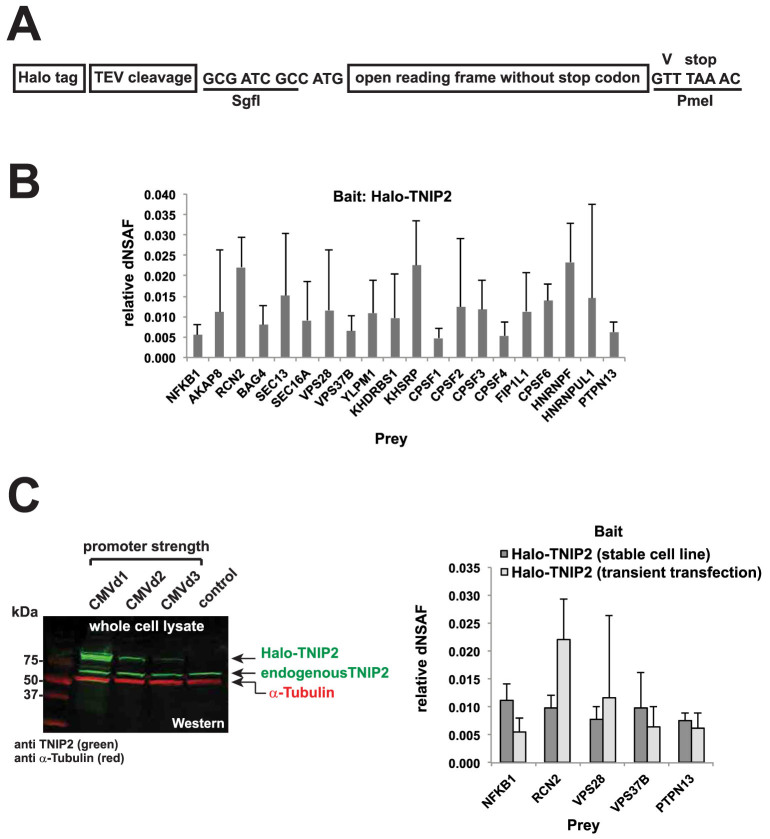
PTPN13 copurifies with Flexi-cloned Halo-TNIP2. (A), the structure of Flexi®-format human ORF clones^10^. Digestion with the restriction enzymes SgfI and PmeI allows the ORF to be subcloned into other suitable vectors. The PmeI site also codes for an additional valine at the C-terminus of each ORF in the library. (B), the top 20 most abundant proteins consistently enriched in samples from cells transiently transfected with Halo-TNIP2 (FDR < 0.01) (see Supplementary Table 1). Results shown have been calculated as described in Methods from 9 biological replicates (Halo tag alone control samples) and 5 biological replicates (Halo-TNIP2 samples). The mean dNSAF values of prey proteins detected in the Halo-TNIP2 samples (normalized to the bait dNSAF) are shown (see Supplementary Table 1). Error bars represent standard deviation. (C), proteins copurifying with Halo-TNIP2 stably expressed at close to endogenous levels. Western blot analysis was used to compare the expression levels of Halo-TNIP2, expressed using different strength promoters, with the expression level of endogenous TNIP2 in HEK293T cells. Purifications using Halo-TNIP2 stably expressed under the control of the CMVd2 promoter were then analysed by mass spectrometry; five of the prey proteins identified in (B) were found consistently enriched (FDR < 0.05) in these samples. Results have been calculated from 4 biological replicates (HEK293T control cells) and 3 biological replicates (stably expressing Halo-TNIP2 cells). The mean dNSAF values of prey proteins detected in the Halo-TNIP2 samples (normalized to the bait dNSAF) are shown. Error bars represent standard deviation.

Previously, we had used Flexi®-format human ORF clones^9^,^10^ encoding various Halo-tagged bait proteins for AP-MS studies investigating the network of protein-protein interactions among members of the NFκB family of transcription factors^11^. The ORF clones are designed with the open reading frame coding for a protein, without the stop codon, flanked by the rare restriction sites SgfI and PmeI (Fig. 1A). Upstream of the SgfI site are sequences coding for the Halo affinity tag and a TEV protease cleavage site (for removal of the tag); downstream and in frame with the ORF, the PmeI restriction site codes for an additional C-terminal valine followed by a stop codon (Fig. 1A). The design enables convenient transfer of the ORFs to other vectors (for example for expression using different strength promoters) by restriction digest with SgfI and PmeI. As cleavage with PmeI (GTTT∧AAAC) produces blunt ends, the excised ORF fragment does not itself code for the stop codon. This allows the ORF to be subcloned into vectors with C-terminal affinity tags if the blunt 3′ end of the ORF is ligated with a blunt end in the destination vector that does not complete the stop codon.

### PTPN13 consistently copurifies with Flexi®-cloned Halo-TNIP2 bait

The 429 aa protein TNIP2 (also known as ABIN-2)^12^ is a known binding partner of NFKB1 (also known as p105)^13^. In order to map protein-protein interactions between recombinant TNIP2 and endogenous cellular proteins, we had used Flexi®-cloned Halo-TNIP2 as a bait for a series of AP-MS experiments. For initial experiments, we transiently transfected HEK293T cells, with a construct using the CMV promoter to express Halo-TNIP2 at relatively high levels. The top 20 most abundant proteins (FDR < 0.01) enriched in purifications using cells transfected with Halo-TNIP2 (compared with control cells expressing the Halo tag alone) are shown in Fig. 1B. As expected we identified the known TNIP2 associated protein NFKB1^13^. Among the other proteins copurifying with TNIP2, we were surprised to find the Fas-associated phosphatase PTPN13 (also known as PTP-BAS or FAP-1)^14^. Although PTPN13 had not previously been reported as a TNIP2 associated factor, PTPN13 had been reported to interact with other components of the TNF/NFκB signaling pathway, including the NFkB inhibitor IkBalpha^15^, and the TNF family receptor Fas^16^. Consequently, to gain additional evidence for what we believed might be a genuine association between TNIP2 and PTPN13, we decided to generate a cell line stably expressing Halo-TNIP2. This time we used a weaker promoter for expressing TNIP2 at close to endogenous levels (Fig. 1C). Both NFKB1 and PTPN13 also copurified with this stably expressed Halo-TNIP2 (Fig. 1C).

### PTPN13 association with Halo-TNIP2 depends on the C-terminal valine “cloning scar”

**Figure 2:**
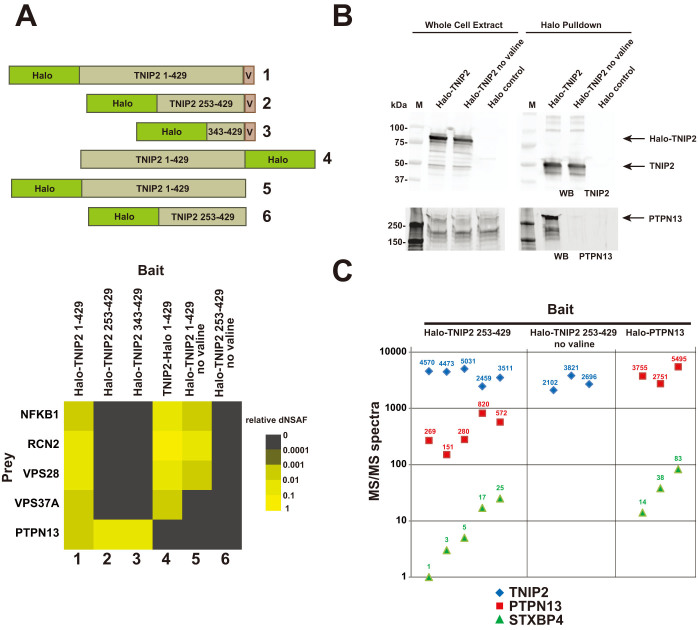
PTPN13 association with Halo-TNIP2 depends on a C-terminal valine cloning scar. (A), a region within the C-terminus of Flexi cloned TNIP2 is important for its association with PTPN13. Plasmids expressing the six Halo-tagged constructs indicated were transiently transfected in HEK293T cells for Halo affinity purification followed by MudPIT mass spectrometry analysis. Relative amounts of the five prey proteins indicated in Figure 1C enriched using each of these six baits (FDR < 0.05) are indicated according to their relative dNSAF value. Average prey dNSAF values were calculated from between three and six replicate experiments for each bait (see Supplementary Table 2). Average prey dNSAF values were then normalized to the average bait dNSAF to generate relative dNSAF values. (B), the association of Flexi-cloned TNIP2 and PTPN13 depends on the C-terminal valine cloning scar. Whole cell extracts from HEK293T cells transfected with the indicated constructs were subjected to Halo affinity chromatography and samples were analysed by SDS-PAGE followed by Western blotting. TNIP2 protein was visualized using rabbit anti-TNIP2 or rabbit anti-PTPN13 primary antibodies, and Alexa-680 labeled anti-rabbit secondary antibodies. Note the change in molecular weight of the TNIP2 bait after purification, which involves removal of the 33 kDa Halo tag. Western blots were imaged using a Li-Cor infra-red imaging system. (C), Halo purified proteins from HEK293T cells transfected with Halo-TNIP2 253–429 (5 biological replicates), Halo-TNIP2 253–429 no valine (3 biological replicates) and Halo-PTPN13 (3 biological replicates) were analysed by mass spectrometry. The numbers of distributed MS/MS spectra corresponding to the proteins TNIP2, PTPN13 and STXBP4 from each of these replicates is indicated. None of these proteins were detected in 9 biological replicates of control purifcations using cells expressing the Halo tag alone.

In order to help us to understand the nature of the association between the two proteins, we decided to determine which regions of Halo-TNIP2 might be important for its association with PTPN13. Consequently, we constructed vectors to express different regions of TNIP2 (Fig. 2A). We detected PTPN13 in purifications using TNIP2 mutants expressing Halo-tagged regions of the C-terminus (amino acids 253–429 or 343–429) suggesting that the C-terminal 87 amino acids of TNIP2 might be important for the association (Fig. 2A columns 2 and 3). We next expressed full length TNIP2 with the Halo tag at the C-terminus to determine whether the position of the tag might affect the TNIP2/PTPN13 association. We did not detect PTPN13 in purifications using C-terminally tagged bait (TNIP2-Halo) (Fig. 2A column 4). This could be the result of steric hindrance by the affinity tag disrupting a genuine TNIP2/PTPN13 interaction. We also considered an alternative possibility. The N-terminally tagged Flexi®-format human ORFeome clones code for an additional valine at the C-terminus of each protein (from the PmeI “cloning scar”). We thought that perhaps the microenvironment created by the combination of the C-terminal amino acids of TNIP2 followed by this additional valine residue might be important for the observed TNIP2/PTPN13 association. To test this, we constructed a vector expressing Halo-TNIP2 without the C-terminal valine usually present in Flexi®-format human ORF clones. When we removed the C-terminal valine “cloning scar” from the Halo-TNIP2 bait, we no longer detected copurifying PTPN13 (Fig. 2A columns 5 and 6 and Fig. 2B). Having observed this loss in TNIP2/PTPN13 association after removing the C-terminal valine, we considered whether additional prey proteins might have been copurifying spuriously with the original Halo-TNIP2 (with the C-terminal valine) via PTPN13. Consistent with this, we noticed that a second protein, STXBP4, was detected in purifications using the bait Halo-TNIP2 253–429 which included the cloning scar valine, but was not in purifications using the same bait but with the valine removed (Fig. 2C). Also in support of STXBP4 copurifying with Halo-TNIP2 via an association with PTPN13, we detected STXBP4 peptides in purifications that used Halo-PTPN13 as bait (Fig. 2C).

### A PTPN13 region containing PDZ domains is sufficient for an interaction with the Halo-TNIP2 bait

**Figure 3:**
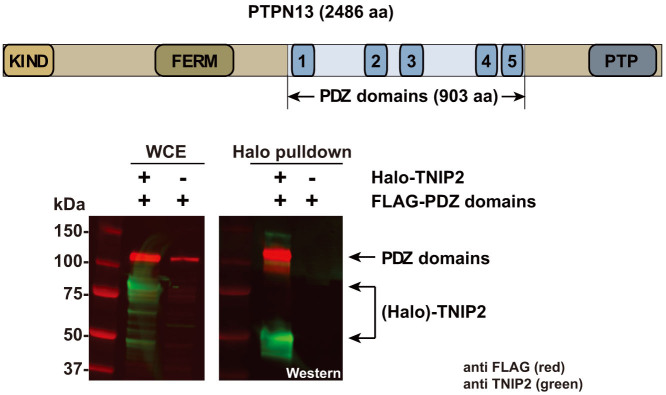
A region of PTPN13 containing the PDZ domains is sufficient for association with Flexi cloned Halo-TNIP2. Part of the PTPN13 ORF coding for a 903 aa region, which included the five PDZ domains, was subcloned into FLAG-pcDNA5/FRT and coexpressed in HEK293T cells with or without Halo-TNIP2 (with the valine cloning scar) as indicated. Lysates were subjected to Halo affinity purification and the resulting eluates analysed by SDS-PAGE and Western blotting. Proteins were detected using anti-FLAG® M2 mouse monoclonal and anti-TNIP2 rabbit polyclonal primary antibodies, and with IRDye® 680LT labeled anti-mouse (red) and IRDye® 800CW anti-rabbit (green) secondary antibodies. Proteins were visualized using a LI-COR® Odyssey® infrared imaging system.

PTPN13 is a 2486 amino acid protein with a number of protein interaction domains^17^ including a KIND module^18^, a FERM domain^19^, and five PDZ domains^20^ (Fig. 3). Notably, PDZ domains often bind peptide motifs at the C-terminus of their interaction partners^21^. Songyang and co-workers had previously screened peptide libraries to investigate peptide-binding specificities of a number of PDZ domains and found a strong preference for a C-terminal valine in the binding motifs for many of the PDZ domains that they studied^22^. As a consequence, we asked whether the region of PTPN13 containing the PDZ domains would copurify with our Halo-TNIP2 protein (which included the additional C-terminal valine) (Fig. 3). Indeed the FLAG-tagged PDZ domain region copurified with Flexi®-format Halo-TNIP2, but was not detected in control purifications. This is consistent with an interaction between the recombinant Halo-TNIP2 and the region of PTPN13 containing the PDZ domains (Fig. 3).

### Spurious association between another Flexi®-format bait, Halo-Jun, and a number of proteins containing PDZ domains

**Figure 4:**
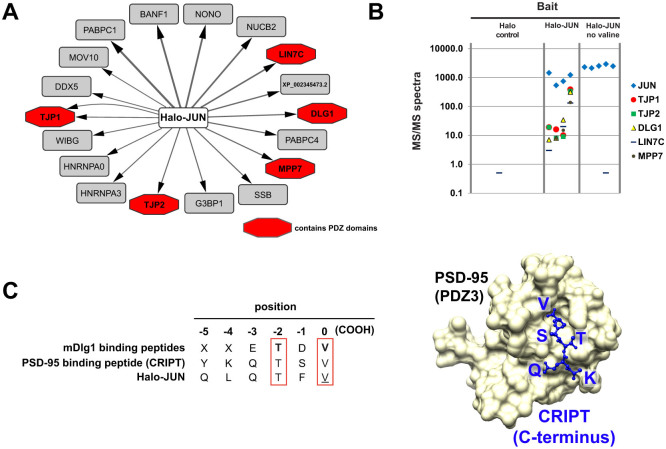
Spurious association between PDZ domain containing proteins and Halo-JUN. (A), top 20 proteins enriched in Halo purified samples (4 biological replicates) from cells expressing Halo-JUN (after contaminant extraction – see Supplementary Table 3). The width of each arrow represents abundance (average dNSAF value). Proteins predicted to contain PDZ domains are shown in red. (B), PDZ containing proteins no longer co-purify with Halo-Jun after removal of the C-terminal valine cloning scar (see Supplementary Table 3). Halo purified proteins from HEK293T cells transfected with Halo-JUN (4 biological replicates), Halo-JUN no valine (5 biological replicates) and Halo-tag (9 biological replicates) were analysed by mass spectrometry. The numbers of distributed MS/MS spectra detected, corresponding to the proteins indicated, is shown for each of these replicates. (C), sequence similarity between PDZ binding motifs, and the sequence of the C-terminus of the Halo-Jun Flexi®-format human ORF clone. The C-terminal 5 amino acids are shown for previously characterized PDZ binding motifs. Motifs binding mDlg1 (PDZ domain 1)^22^ and PSD-95^23^ are shown. The structure of the PDZ3 domain of PSD-95 complexed with a peptide (KQTSV) corresponding to the C-terminus of CRIPT (blue) [PDB accession number 1BE9]^23^ is shown on the right.

The spurious association between Halo-TNIP2 and endogenous PTPN13 could have been an isolated example of a false positive interaction due to the cloning scar valine at the C terminus of the bait protein. Alternatively some of the other Flexi®-format bait proteins that we had used might similarly have interacted speciously with proteins containing PDZ domains. We had previously used ~30 Flexi®-format human ORF clones to express Halo-tagged baits for AP-MS studies. Of these, we noticed that with Flexi-cloned Halo-JUN used as bait, five proteins that had been previously annotated as containing PDZ domains were among the most significant prey proteins identified (Fig. 4A). These included two isoforms of the tight junction protein TJP1 and its paralog TJP2, MPP7, LIN7C, and the protein encoded by DLG1, a human homolog of the *Drosophila melanogaster* gene discs large 1. These proteins failed to copurify with Halo-JUN lacking the C-terminal valine cloning scar (Fig. 4B). Interestingly, a GST fusion using the first PDZ domain within the mouse homolog of DLG1 (mDlg-1) had been previously used to search a peptide library to determine a consensus peptide binding motif for this PDZ domain^22^. We compared this consensus sequence with the C terminus of the Flexi®-format Halo-JUN bait (Fig. 4B). The consensus sequence determined by Songyang and coworkers contains a strong preference for the amino acids threonine and valine at positions −2 and 0 respectively; similarly, our recombinant Halo-Jun bait contains a threonine at position −2, and a valine from the PmeI cloning scar at 0. In a different study, Doyle and co-workers had determined the crystal structure of the third PDZ domain of rat PSD-95 (also known as DLG4) in a complex with a peptide corresponding to the C-terminus of CRIPT, a protein they had identified as a putative binding partner^23^(Fig. 4C). We noted the curious similarity between the sequence of the C-terminus of this PDZ domain binding peptide (QTSV) and the C-terminus of Flexi®-format Halo-Jun (QTFV). (Fig. 4C).

## Discussion

Following the sequencing of the human genome and the subsequent technological advances in the field of genomics, more recent efforts have focused on defining the interactome, the network of dynamic protein-protein interactions that occurs in cells^2^. One of the approaches taken in large scale proteomics studies makes use of protein expression libraries containing collections of ORF clones^6^; these can be used for expressing a variety of bait proteins in cells. These baits are then used to prepare protein complexes that can be analysed by mass spectrometry^24^, enabling the network of interactions between cellular proteins to be mapped^25^. In this report, we have identified a source for false positive protein-protein interactions identified in such proteomics studies. Specifically, we have found that an additional valine encoded at the C-terminus of protein coding sequences in a commercially available human ORF library^26^ can sometimes result in spurious binding of specific endogenous cellular proteins to the recombinant bait protein. The aberrant interactions depend on the combination of: 1) the C-terminal amino acid sequence of the native version of the bait protein, and 2) the additional C-terminal valine appended to the C-terminus of the recombinant protein. Because the resulting artificial binding motifs result from this combination, their occurrence will vary from one bait to another and so are difficult to detect using conventional controls. Although the aberrant interactions that we have found result from this single amino acid added to the C-terminus, it is possible that similar spurious binding events that are not easily controlled for may occur in other systems using recombinant baits which are modified versions of the endogenous protein. For example, Wissmueller and co-workers found that a GST tag added to the KLF3 protein caused misfolding of the KLF3 which resulted in spurious binding between KLF3 and GATA-1^27^. Whether the additional amino acid sequences that are added are short affinity tags or sequences to facilitate the transfer of ORFs between vectors (restriction or recombination sites), if cryptic protein binding sites are created that rely on the combination of these fixed sequences and the variable sequences within the ORFs, any resulting false positive interactions may not be detected using simple experimental controls.

Initially, our experiments aimed to begin to define the network of protein-protein interactions between members of the NFκB and AP-1 families of transcription factors in cells. The approach that we took, using affinity tagged baits from a commercially available ORFeome collection to purify protein complexes and identify their components, has been used extensively for interactome mapping studies^8^,^28^,^29^. Using Halo-TNIP2 as bait, our early studies appeared to have identified the protein PTPN13 as a novel TNIP2 associated protein. To avoid artefacts caused by overexpression of baits and for increased confidence in the veracity of a result, Gibson et al. have suggested that initial experiments using transiently overexpressed proteins are confirmed using approaches using engineered cell lines expressing tagged proteins at close to native levels^30^. We found that the association between TNIP2 and PTPN13 was suggested both in experiments using transiently transfected cells overexpressing tagged TNIP2, and in experiments using cells stably expressing the TNIP2 bait at close to endogenous levels (Fig. 1). It was only when we were in the process of conducting more detailed follow-up studies, so that we could more closely define the region of the bait needed for the Halo-TNIP2/PTPN13 association, that the cause of the association became apparent. The additional valine, which had been appended to the C-terminus of all ORFs in the library, had resulted in the presence of PTPN13 in the Halo-TNIP2 purifications (Fig. 2A and 2B). PTPN13 was not detected in control purifications (using cells expressing the Halo affinity tag alone), or in purifications using other Halo tagged bait proteins (that similarly had an additional C-terminal valine). In addition to the false positive identification of PTPN13, the spurious purification of PTPN13 with TNIP2 likely resulted in a secondary false positive copurifying protein, STXBP4 (Fig. 2C). After examining both the sequence of the C-terminus of the Halo-TNIP2 ORF, as well as the predicted protein interaction domains in PTPN13 (Fig. 3A), we thought that the association might result from an artificial PDZ binding motif created at the C terminus of the Flexi®-format human ORF. In support of this, we found that a region of PTPN13 containing the PDZ domains (and not the other annotated protein binding domains) copurified with affinity purified Halo-TNIP2 (Fig. 3). Once we had identified the modified C terminus of TNIP2 as the likely source of its association with PTPN13, we considered whether PDZ domain containing proteins might have spuriously copurified with any of the other baits we had used in our study. Of the ~30 baits that we had used, we found a second example in which a number of proteins with predicted PDZ domains copurified with Flexi®-format Halo-JUN (Fig. 4). As with Halo-TNIP2, the proteins with PDZ domains did not copurify with the bait once the C-terminal valine “cloning scar” had been removed. Again we thought that these associations might result from the C-terminus of the Flexi®-format Halo-JUN construct binding to the hydrophobic cleft in the PDZ domains in the prey proteins. Supporting this idea, when we examined the sequence of Halo-JUN, we found that it closely matched the sequences of known PDZ domain binding motifs^22^,^23^,^31^(Fig. 4C).

There has been an increasing awareness of the importance of the reproducibility of scientific findings, both for science research in general^32^,^33^,^34^,^35^, and for the field of proteomics in particular^36^,^37^. Many false-positive findings may well have obscure technical causes (examples include misfolding of the bait protein due to the addition of an affinity tag or due to the presence of EDTA^27^,^38^,^39^). Here, we have identified one such technical issue through careful follow-up studies on an apparently novel interaction. Similar issues in which false–positive results are not easily revealed through common control experiments may exist in other systems; careful and extensive follow-up studies are essential in validating seemingly novel protein-protein interactions.

## Methods

### Materials

Mouse anti-α-Tubulin (T9026) and mouse anti-FLAG® M2 (F3165) monoclonal antibodies were purchased from Sigma. Rabbit anti-TNIP2 (NBP1-32689) and rabbit anti-PTPN13 (NB100-56139) polyclonal antibodies were from Novus Biologicals. IRDye® 800CW labeled goat anti-Rabbit (926–3211) and IRDye® 680LT labeled goat anti-Mouse (926–68020) secondary antibodies were from LI-COR Biosciences (Lincoln, NE). Human ORF clones Halo-TNIP2 (FHC21846) and JUN (FXC01532) were from the Kazusa DNA Research Institute (Kisarazu, Chiba, Japan). Halo-PTPN13 (EX-E2580-M49) was from GeneCopoeia (Rockville, MD). Magne™ HaloTag® magnetic affinity beads were from Promega (Madison, WI).

### Cell culture

HEK293T cells (ATCC® CRL-11268™) were from ATCC (Manassas, VA) and Flp-In™-293 cells (AHO1112) were from Invitrogen™ (Carlsbad, CA). HEK293T cells (testing mycoplasma negative: Jan 13^th^ 2009) and Flp-In™-293 cells (testing mycoplasma negative: Jun 2^nd^ 2008) are stocked in a master cell bank with a new vial thawed every ~30 passages. Cells from the master stock are also tested randomly for mycoplasma when they are in passage using the Universal Mycoplasma Detection Kit (ATCC® 30-1012K). Both HEK293T and Flp-In™-293 cell lines were most recently authenticated by STR profiling using the Cell Line Authentication Service (Promega) on 26^th^ June 2014.

### Subcloning FLAG® and Halo®- tagged bait proteins

The TNIP2 ORF was subcloned into different Flexi® vectors as described by Blommel et al.^40^. In brief, the ORF was transferred from the original Flexi® vector (pFN21A – used for Halo-tagged protein expression driven by the CMV promoter) into the Flexi® vectors pFN22A, pFN23A and pFN24A (for Halo-tagged protein expression in transiently transfected cells using progressively weaker promoters, Fig. 1C) or into pFC14A (for expressing proteins with a C-terminal Halo® tag, Fig. 2A). Halo-JUN was contructed by transferring the ORF coding for JUN from the original Flexi® vector (pF1K) into the vector “Halo pcDNA5/FRT PacI PmeI” (described in Banks et al.^11^). For stable expression of TNIP2, we constructed the vector “CMVd2 Halo pcDNA5/FRT PacI PmeI” by inserting a DNA fragment containing the CMVd2 promoter followed by a sequence coding for the Halo® tag between the MluI and KpnI restriction sites of the vector pcDNA5/FRT (Invitrogen™). Using the vector pFN23A as a template, this DNA fragment was synthesized using the primers: MluI CMVd2 fwd (5′-CAGACGCGTGACGCAAATGGGCGGTAGGC-3′) and KpnI PacI Halo rev (5′-CAGGGTACCTTAATTAAGTTATCGCTCTGAAAGTACAGATCCTCAGTGG-3′). Sequences of the primers used to construct the vectors used to express the Halo tagged C-terminal regions of TNIP2 (Fig. 2A) or to remove the C-terminal valine from Halo-TNIP2 or Halo-JUN, are given in Supplementary Table 4.

### Preparation of Whole Cell Extracts

For experiments using transiently transfected cells, extracts from approximately 2 × 10^7^ HEK293T cells transfected with 7.5 μg plasmid DNA encoding the proteins indicated in the figures were prepared as described^11^. For the experiment described in Figure 1C, Flp-In™-293 cells stably expressing Halo-TNIP2 under the control of the CMVd2 promoter were generated using the Flp-In™ system (Invitrogen™) according to the manufacturer's instructions. For each experiment, dishes containing either 1 × 10^8^ Halo-TNIP2 expressing cells, or HEK293T cells (control) were cultured for 72 hours. Cells were harvested and washed twice in ice-cold PBS. Cell pellets were incubated at −80°C for 30 minutes, thawed, and resuspended in 1 ml of ice-cold buffer containing 50 mM Tris·HCl (pH 7.5), 150 mM NaCl, 1% Triton® X − 100, 0.1% sodium deoxycholate, 0.1 mM benzamidine HCl, 55 μl phenanthroline, 10 μM bestatin, 20 μM leupeptin, 5 μM pepstatin A, and 1 mM PMSF. Lysates were then passed through a 26-gauge needle five times. To remove insoluble material, homogenized samples were centrifuged at 21,000 × g for 30 minutes.

### Halo® affinity chromatography

Either 1 ml whole cell extract (stably expressing cells), or 300 μl of whole cell extract diluted with 700 μl TBS (transiently transfected cells) was used for purifying Halo-tagged bait complexes using Magne™HaloTag® magnetic affinity beads (Promega). The extracts were incubated for 1 hour at 4° with beads prepared from 100 μl bead slurry. The beads were washed four times in buffer containing 50 mM Tris·HCl (pH 7.4), 137 mM NaCl, 2.7 mM KCl, and 0.05% Nonidet®P40. Bound proteins were eluted by incubating the beads for 2 h at 25°C in 100 μl buffer containing 50 mM Tris·HCl (pH 8.0), 0.5 mM EDTA, 0.005 mM DTT, and 2 units of AcTEV™ Protease (Invitrogen). To remove any traces of affinity resin, the eluates were spun through Micro Bio-Spin® columns (BioRad).

### Mass spectrometry

Halo-purified proteins were precipitated with trichloroacetic acid and centrifuged at 21,000 × g for 30 minutes at 4°C. The resulting pellet was washed twice with acetone and resuspended in buffer containing 100 mM Tris·HCl (pH 8.5) and 8 M urea. The sample was treated with Tris(2-carboxylethyl)-phosphine hydrochloride to reduce disulphide bonds, chloroacetamide (to prevent bond reformation), and digested with endoproteinase Lys-C for 6 hours at 37°C. Samples were digested overnight with trypsin as described previously^41^. The resulting peptides were resolved using MudPIT mass spectrometry as described previously^11^. In brief, peptides were resolved using three-phase microcapillary columns and gradually eluted into an LTQ mass spectrometer (Thermo Scientific) over a period of approximately 20 hours.

### Analysis of Mass Spectrometry data

Mass spectrometry data was analysed essentially as described previously^11^. Following mass spectrometry, raw files were processed using an in-house software package (RAWDistiller v. 1.0) to generate ms2 files. The SEQUEST algorithm (version 27, rev. 9) was used to match MS/MS spectra to 29,375 human protein sequences (National Center of Biotechnology Information, November 2010 release)^42^. DTASelect was used remove matches with parameters below selected threshold values^43^. Filtering parameters included: minimum XCorr value of 1.8 (singly charged spectra), 2.5 (doubly charged spectra), and 3.5 (triply charged spectra); minimum DeltCn value of 0.08; maximum Sp rank of 10; and a minimum peptide length of 7 amino acids. We used a minimum of three biological replicates of each type of sample for analysis. Replicates were excluded from analysis when the MudPIT mass spectrometry run failed (4 samples), or when the fewer than 500 MS/MS spectra corresponding to the bait protein were detected (2 samples). MudPIT run failure can occur when the microcapillary column becomes clogged. For the analysis of data in Figure 1, Figure 2A and Figure 4A, the abundance of proteins identified in more than half of the replicate experimental samples was quantified using spectral counting to calculate dNSAF values using Contrast and NSAF7 software^43^,^44^. Proteins with a high probability of being enriched in experimental samples relative to control samples were determined using the PLGEM algorithm^45^ (a number of the controls used here were also used as part of an earlier study^11^). To adjust for multiple comparisons, false discovery rates (FDRs) were calculated from PLGM p-values using the method of Benjamini and Hochberg^46^. For the analysis of data in Figure 2C and 4B, NSAF7 was used to determine the number of distributed spectra detected in each sample corresponding to the subset of proteins shown. Mass spectrometry data sets have been deposited to the PeptideAtlas repository^47^ (www.peptideatlas.org) with the identifiers PASS00598 to PASS00609 and password GZ5438hrm.

## Author Contributions

C.A.S.B., G.B., Z.T.L. and M.P.W. designed experiments, C.A.S.B., G.B. and Z.T.L. collected and analysed the data. L.F. contributed analytic tools. C.A.S.B. and M.P.W. wrote the manuscript.

## Supplementary Material

Supplementary Information Supplementary Table 1

Supplementary Information Supplementary Table 2

Supplementary Information Supplementary Table 3

Supplementary Information Supplementary Table 4
